# ZeMYB9 regulates cyanidin synthesis by activating the expression of flavonoid 3′-hydroxylase gene in *Zinnia elegans*

**DOI:** 10.3389/fpls.2022.981086

**Published:** 2022-10-18

**Authors:** Jieyu Qian, Lingli Jiang, Hongsheng Qing, Jiahong Chen, Ziyun Wan, Menghan Xu, Jianxin Fu, Chao Zhang

**Affiliations:** ^1^ Zhejiang Provincial Key Laboratory of Germplasm Innovation and Utilization for Garden Plants, Zhejiang Agriculture & Forestry University, Hangzhou, China; ^2^ Key Laboratory of National Forestry and Grassland Administration on Germplasm Innovation and Utilization for Southern Garden Plants, Zhejiang Agriculture & Forestry University, Hangzhou, China; ^3^ School of Landscape Architecture, Zhejiang Agriculture & Forestry University, Hangzhou, China

**Keywords:** *Zinnia elegans*, anthocyanin, flavonoid 3′-hydroxylase, MYB, bHLH

## Abstract

Petal color in *Zinnia elegans* is characterized mainly by anthocyanin accumulation. The difference in the content of anthocyanins, especially cyanidins, affects petal coloration in *Z. elegans*, but the underlying regulatory mechanism remains elusive. Here, we report one R2R3-MYB transcription factor from subgroup 6, ZeMYB9, acting as a positive regulator of anthocyanin accumulation in *Z. elegans*. Up-regulated expression of *ZeMYB9* and flavonoid 3′-hydroxylase gene (*ZeF3’H*) was detected in the cultivar with higher cyanidin content. ZeMYB9 could specifically activate the promoter of *ZeF3’H*, and over-expression of *ZeMYB9* induces much greater anthocyanin accumulation and higher expression level of anthocyanin biosynthetic genes in both petunia and tobacco. And then, ZeMYB9 was demonstrated to interact with ZeGL3, a bHLH transcription factor belonging to IIIf subgroup. Promoter activity of *ZeF3’H* was significantly promoted by co-expressing *ZeMYB9* and *ZeGL3* compared with expressing *ZeMYB9* alone. Moreover, transient co-expression of *ZeMYB9* and *ZeGL3* induced anthocyanin accumulation in tobacco leaves. Our results suggest that ZeMYB9 could enhance cyanidin synthesis and regulate petal color in *Z. elegans* though activating the expression of *ZeF3’H*, by itself or interacting with ZeGL3.

## Introduction

Flower color, one of the most important ornamental traits for floricultural crops, is mainly due to the accumulation of three kinds of pigments: flavonoids, carotenoids and betalains (Tanaka et al., 2008). Anthocyanins, belonging to a colored class of flavonoids, make flowers with a diverse range of hues from orange, red, to blue and violet (Tanaka et al., 2008). Anthocyanin synthesis is relatively conserved in higher plants, mainly based on a series of catalytic enzymes including chalcone synthase (CHS), chalcone isomerase (CHI), flavanone 3-hydroxylase (F3H), flavonoid 3′-hydroxylase (F3′H), dihydroflavonol 4-reductase (DFR), anthocyanidin synthase (ANS), anthocyanin glucosyltransferase (GT), anthocyanin acyltransferase (AT) and methyltransferase (MT) (Tanaka et al., 2010).

The number of hydroxyl groups on the B-ring of anthocyanin is one of the essential factors affecting the color of anthocyanin pigments. Blue/violet flowers are inclined to accumulate trihydroxylated delphinidin-based anthocyanins, red/magenta flowers tend to contain cyanidin-based anthocyanins (with two hydroxyl groups on the B-ring), while orange/brick red flowers predominantly contain pelargonidin-based anthocyanins with only one hydroxyl group on the B-ring (Tanaka and Brugliera, 2013).

Two cytochromes P450, F3′H and F3′5′H play a crucial role in the determination of flower color and anthocyanin profile by introducing hydroxyl groups to the B-ring (Tanaka, 2006). Flowers could accumulate blue-flower-specific anthocyanidins, such as delphinidin, petunidin and malvidin, which is due to the presence of F3′5′H activity (Shimada et al., 2014). However, many ornamental plants, such as rose (*Rosa hybrida*), chrysanthemum (*Chrysanthemum morifolium*), carnation (*Dianthus caryophyllus*), naturally lack F3′5′H activity, so they cannot synthesize delphinidin-based anthocyanins, which leads to the deficiency of violet/blue flower colors in these plant species (Holton et al., 1993). The introduction of heterologous *F3′5′H* gene into these plants, results in novel purple/violet flowers owing to the formation and accumulation of delphinidin-based anthocyanins (Fukui et al., 2003; Katsumoto et al., 2007; Noda et al., 2017). Another cytochrome P450 F3′H can catalyze the hydroxylation at the 3′-position of the B-ring and convert dihydrokaempferol (DHK) into dihydroquercitin (DHQ), resulting in a metabolic flux shift toward cyanidin biosynthesis and the production of cyanidin-based anthocyanins (Tanaka and Brugliera, 2013). Therefore, spontaneous mutations of *F3′H* gene in the morning glory species (*Ipomoea nil*, *I. purpurea* and *I. tricolor*) lead to the change of flower color and redirection of the anthocyanin biosynthesis pathway from cyanidin into pelargonidin (Hoshino et al., 2003). In addition, the expression level of *F3′H* gene was closely associated with the anthocyanin composition and content in the fruits of different *Fragaria* species. *F.* × *ananassa* fruits with lower expression of *F3′H* showed a prevalence of pelargonidin, while *F. vesca* with up-regulated expression of *F3′H* had a higher content of cyanidin (Thill et al., 2013).

In addition to catalytic enzyme genes, anthocyanin biosynthesis is also controlled by three types of transcription factors: MYB, bHLH (basic helix-loop-helix) and WD repeat (WDR) (Albert et al., 2014; Xu et al., 2015; Zhou et al., 2019). In plants, MYB transcription factors could be divided into four main types, and the number of R2R3 type MYBs is the largest (Yanhui et al., 2006). Many plant R2R3-MYBs are reported as the key factors in regulating anthocyanin biosynthesis (LaFountain and Yuan, 2021; Yan et al., 2021), of which subgroup 6 (SG6) R2R3-MYBs act as activators of anthocyanin biosynthesis (Quattrocchio et al., 1999; Jian et al., 2019; Fang et al., 2021). Overexpression of *AtMYB75*/*PAP1*, *AtMYB90*/*PAP2*, *AtMYB113* or *AtMYB114* of SG6 promotes anthocyanin accumulation in *Arabidopsis* (Teng et al., 2005; Gonzalez et al., 2008). SG6 R2R3-MYB alone or MYB-bHLH-WDR (MBW) complex enhance anthocyanin accumulation *via* binding to a specific *cis*-acting element (C/TAACG/TG) to activate the expression of structural genes involved in anthocyanin synthesis (Gonzalez et al., 2008). In *Chrysanthemum morifolium*, *CmMYB6* directly activates the expression of *CmDFR*, which could be enhanced when CmMYB6 interacts with a IIIf bHLH protein CmbHLH2 to form a transcriptional complex (Xiang et al., 2015; Xu et al., 2015). Similarly, AcMYBF110 alone or in interaction with AcbHLH1, AcbHLH4, or AcbHLH could regulate the expression of *AcCHS, AcF3′H, AcANS, AcUFGT3a, AcUFGT6b, and AcGST1* in kiwifruit (*Actinidia chinensis*) (Liu et al., 2021).

*Zinnia elegans*, a popular ornamental plant, is rich in flower color and anthocyanins are the main pigments in the petals of this species (Qian et al., 2021). Four main anthocyanins, including cyanidin 3-O-(6′′-acetyl) glucoside-5-O-glucoside (Cy3AG5G), cyanidin 3-O-(6′′-acetyl)glucoside (Cy3AG), pelargonidin 3-O-(6′′-acetyl)glucoside-5-O-glucoside (Pg3AG5G) and pelargonidin 3-O-(6′′-acetyl) glucoside (Pg3AG), have been identified in previous studies (Qian et al., 2019; Qian et al., 2021). Anthocyanin profile is quite different in different color cultivars of *Z. elegans*. Pink and coral cultivars show a prevalence of pelargonidins, whereas cyanidins account for more than half of the total anthocyanins in purple and red cultivars (Qian et al., 2021). In addition, the expression levels of *ZeF3′H* gene are greatly higher in purple and red cultivars than those in pink and coral cultivars (Qian et al., 2021), suggesting a close link between *ZeF3′H* expression and cyanidin accumulation in *Z. elegans*. Among three identified SG6 R2R3-MYBs based on the petal transcriptome data, the expression level of *ZeMYB9* was significantly correlated with that of *ZeF3’H* during petal development in red cultivar of *Z. elegans* (Qian et al., 2022). At present, how ZeMYB9 functions between different color cultivars of *Z. elegans* is not clear. What’s more, the underlying regulatory mechanism by which ZeMYB9 activates the expression of *ZeF3′H* and enhances cyanidin biosynthesis remains unknown, and should be further addressed.

In this study, anthocyanin content and the expression pattern of *ZeMYB9* and *ZeF3’H* were analyzed between pink (lower cyanidin accumulation) and purple (higher cyanidin accumulation) cultivars. Next, yeast one-hybrid and dual luciferase assays were utilized to investigate the interaction between ZeMYB9 and the promoter of the target gene *ZeF3’H*. Using transient assays in petunia flower and stable transformation in tobacco, ZeMYB9 was demonstrated to be a positive regulator of anthocyanin biosynthesis. Furthermore, the interaction between ZeMYB9 and a IIIf bHLH protein ZeGL3, identified by yeast two-hybrid, bimolecular fluorescence complementation and luciferase complementary assays, greatly enhanced the expression of *ZeF3’H* than ZeMYB9 alone did, and also resulted in abundant anthocyanin accumulation in tobacco leaves. Our results revealed that ZeMYB9 was responsible for the difference in anthocyanin profile and petal color of different color cultivars in *Z. elegans*, which will provide insights into the regulatory mechanism of anthocyanin biosynthesis and petal color development in *Z. elegans*.

## Results

### Anthocyanin and petal color analyses

**Figure 1:**
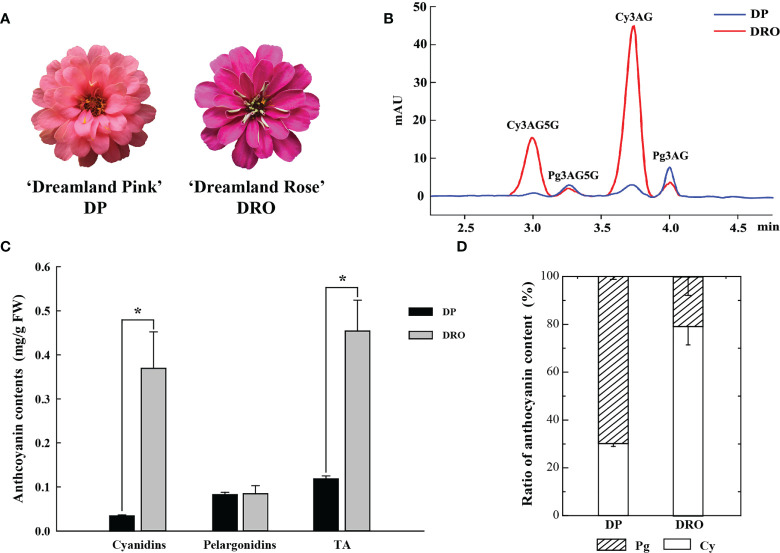
The content of anthocyanin components in two cultivars. **(A)** Inflorescences of *Zinnia elegans* ‘Dreamland Pink’ (DP) and ‘Dreamland Rose’ (DRO) at the full opening stage (S4). **(B)** UPLC chromatogram of DP and DRO at 530nm. **(C)** The comparison of the content of cyanidins, pelargonidins and total anthocyanin (TA) between DP and DRO. **(D)** Proportion analysis of the anthocyanin component contents in DP and DRO. Error bars indicated standard error (SE) of three replicates. T-test was used for statistical analyses between two cultivars (*P < 0.05).

Similar to our previous study (Qian et al., 2021), four anthocyanins were detected in ‘Dreamland Pink’ (DP) with pink petal color and ‘Dreamland Rose’ (DRO) with purple petal color (**Figure 1A**), including cyanidin 3-O-(6′′-acetyl) glucoside-5-O-glucoside (Cy3AG5G), cyanidin 3-O-(6′′-acetyl)glucoside (Cy3AG), pelargonidin 3-O-(6′′-acetyl)glucoside-5-O-glucoside (Pg3AG5G) and pelargonidin 3-O-(6′′-acetyl) glucoside (Pg3AG) (**Figure 1B**). There was no difference in the content of pelargonidins between these two cultivars, but the content of cyanidins in DRO was significantly higher than that of DP (0.37 mg/g FW and 0.04 mg/g FW, respectively), which resulted in markedly higher total anthocyanin content (TA) in DRO (**Figure 1C**). Cyanidins in DRO accounted for 79.3% of TA, which was more than twice as much as that in DP (**Figure 1D**). In addition, there was no significant difference in total flavonoids contents between these two cultivars (**Figure S1A**). Based on petal color analysis, DRO exhibited higher a* and C* values, and lower L*, b* and *h°* values, compared with DP (**Figure S1B**). These results suggested that cyanidin content should be responsible for the color difference between these two cultivars.

### Expression analysis of *ZeF3’H* and *ZeMYB9* and sequence analysis of ZeMYB9

**Figure 2:**
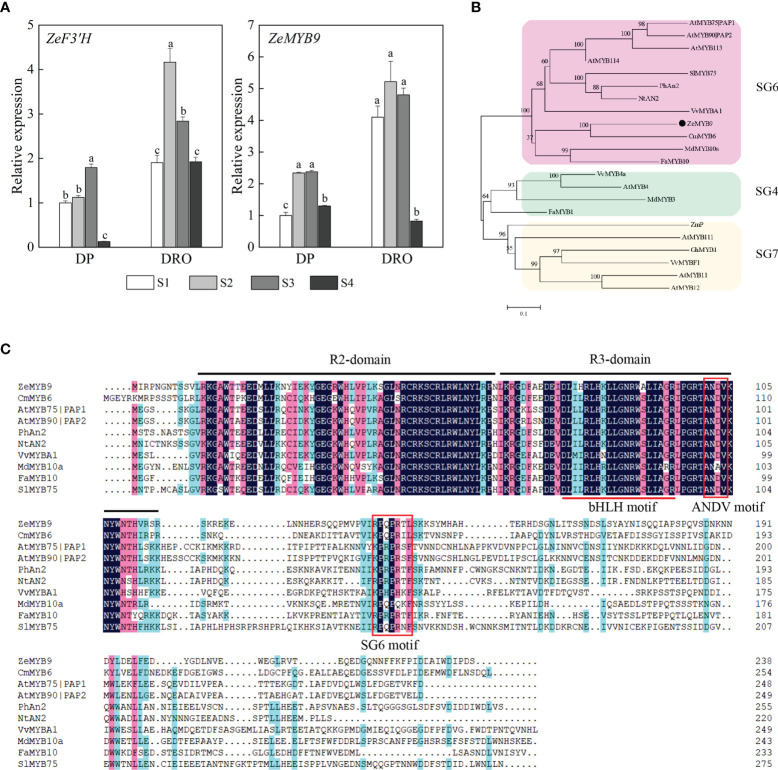
Expression analysis of *ZeF3’H* and *ZeMYB9* and sequence analysis of ZeMYB9. **(A)** Relative expression levels of *ZeF3’H* and *ZeMYB9* in the petals of DP and DRO at different developmental stages. *ZeACT* gene was used as an internal control for normalization, and three biological replicates were performed. Error bars indicated standard error (SE). And different lowercases indicate significant differences (*P* < 0.05). **(B)** Phylogenetic tree of different R2R3-MYB proteins participating in anthocyanin biosynthesis in other plants. **(C)** Multiple alignment of the amino acid sequences of SG6 R2R3-MYB proteins from different plants. The black line at the top indicated the conserved R2-domain and R3-domain. The red underline indicated a motif ([D/E]Lx_2_[R/K]x_3_Lx_6_Lx_3_R) interacting with a bHLH transcription factor. The red box indicated the conserved ANDV motif and SG6 motif. The GenBank accession numbers of MYB proteins from other species were as follows: the SG6 MYBs as *Arabidopsis thaliana* AtMYB75|PAP1 (AT1G56650.1), *A. thaliana* AtMYB90|PAP2 (AT1G66390.1), *A. thaliana* AtMYB113 (AT1G66370.1), *A. thaliana* AtMYB114 (AT1G66380.1), *Solanum lycopersicum* SlMYB75 (NP_001265992.1), *Petunia hybrida* PhAn2 (AAF66727.1), *Nicotiana tabacum* NtAN2 (ACO52470.1), *Vitis vinifera* VvMYBA1 (BAD18977.1), *Chrysanthemum morifolium* CmMYB6 (KR002097.1), *Malus domestica* MdMYB10a (ABB84753.1), *Fragaria ananassa* FaMYB10 (ABX79947.1); the SG4 MYBs as *V. vinifera* VvMYB4a (NP_001268129.1), *A. thaliana* AtMYB4 (AT4G38620.1), *M. domestica* MdMYB3 (AEX08668.1), *F. ananassa* FaMYB1 (AAK84064.1); the SG7 MYBs as *Zea mays* ZmP (P27898), *A. thaliana* AtMYB11 (AT3G62610.1), *A. thaliana* AtMYB12 (AT2G47460.1), *A. thaliana* AtMYB111 (AT5G49330.1), *Gerbera hybrid* GhMYB1 (CAD87007), *V. vinifera* VvMYBF1 (ACT88298).

*ZeF3’H*, a homologous gene of *F3’H* essential for cyanidin biosynthesis, showed significantly higher expression level in DRO than that in DP at the corresponding stage (**Figure 2A**). A R2R3-MYB protein ZeMYB9 was considered as the potential regulatory factor of *ZeF3’H* (Qian et al., 2022), and the expression level of *ZeMYB9* was also greatly higher in DRO (**Figure 2A**), which was highly correlated with the expression level of *ZeF3’H*. Phylogenetic analysis showed that ZeMYB9 was grouped into SG6 and closely related to CmMYB6 (GenBank Accession Number KR002097.1) from *Chrysanthemum morifolium* (**Figure 2B**). Multiple sequence alignment of ZeMYB9 and other SG6 MYB proteins (**Figure 2C**) revealed that the conserved R2 and R3 domains were at the N-termina, and a conserved bHLH motif interacting with a bHLH transcription factor and suitable identifier ANDV were all present in the R3 domain. In addition, ZeMYB9 also had SG6 motif, which was identified to positively regulate anthocyanin synthesis (Rodrigues et al., 2021).

### ZeMYB9 could directly bind to and activate the promoter of *ZeF3’H*

**Figure 3:**
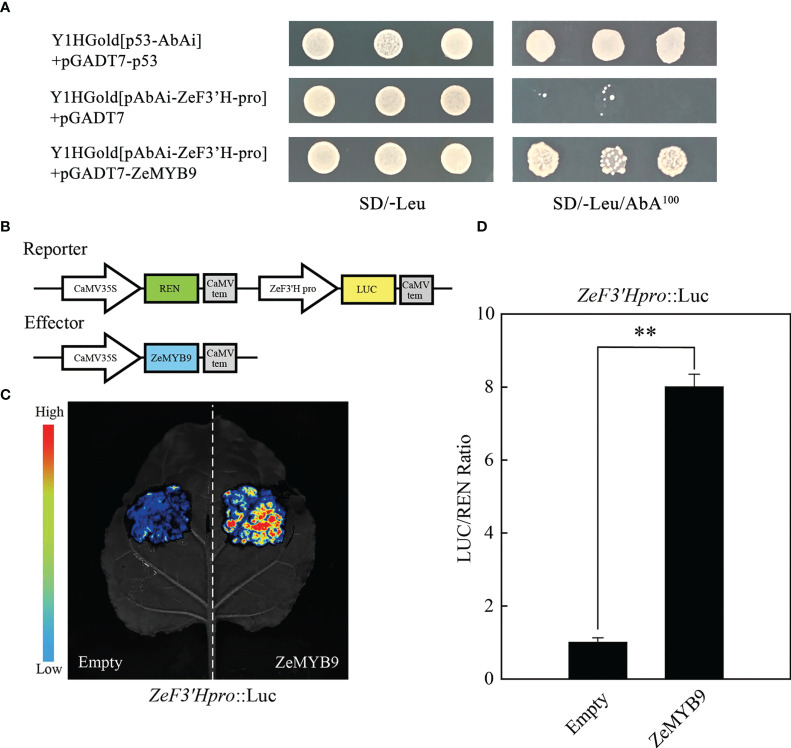
ZeMYB9 could directly bind to the promoter of *ZeF3’H*. **(A)** Yeast one-hybrid assay showing the binding of ZeMYB9 to the promoter of *ZeF3’H*. **(B)** Construction diagrams of reporter and effector vectors for dual-luciferase assays. **(C)** Graph showing the luminescence intensity of luciferase in *N. benthamiana* leaves. **(D)** Transient dual-luciferase detections of *ZeF3’H* in *N. benthamiana* leaves. Data were showed as the mean ± SE from three biological replicates. T-test was used for statistical analyses compared with corresponding control (**P < 0.01).

A 1703 bp sequence (*ZeF3’H_pro_*) upstream of the initiation codon of *ZeF3’H* was isolated, which contained four MYB elements. To identify whether ZeMYB9 can directly bind to the promoter of *ZeF3’H*, yeast one-hybrid and dual luciferase assay were carried out. Co-transformed yeasts of pGADT7-ZeMYB9 and pAbAi-ZeF3’H-Pro could survive and grow normally on the selective medium of SD/-Leu/100 ng mL^−1^ AbA, but the negative control did not survive (**Figure 3A**), revealing that ZeMYB9 can interact with *ZeF3’H* promoter in the yeast system. In addition, a dual luciferase assay was employed to further verify the regulation of ZeMYB9 on the *ZeF3’H* promoter. The reporter plasmid contained *LUC* and *REN* expressed under the control of *ZeF3’H*promoter and 35S promoter, respectively, while the effector vector carried *ZeMYB9* under the control of 35S promoter (**Figure 3B**). *ZeF3’H_pro_*::LUC were transiently co-transformed with 35S::ZeMYB9 or 35S::empty into *N. benthamiana* leaves. Signal of LUC was significantly increased when *ZeF3’H_pro_*::LUC reporter was co-transformed with 35S::ZeMYB9 effector compared with 35S::empty effector (**Figure 3C**). And the LUC/REN ratio with 35S::ZeMYB9 effector was eight times higher than the control (**Figure 3D**). The results demonstrated that ZeMYB9 could bind to promoter and positively regulate the expression of *ZeF3’H*.

### Heterologous expression of *ZeMYB9* strongly promoted anthocyanin accumulation in petunia and tobacco flowers

**Figure 4:**
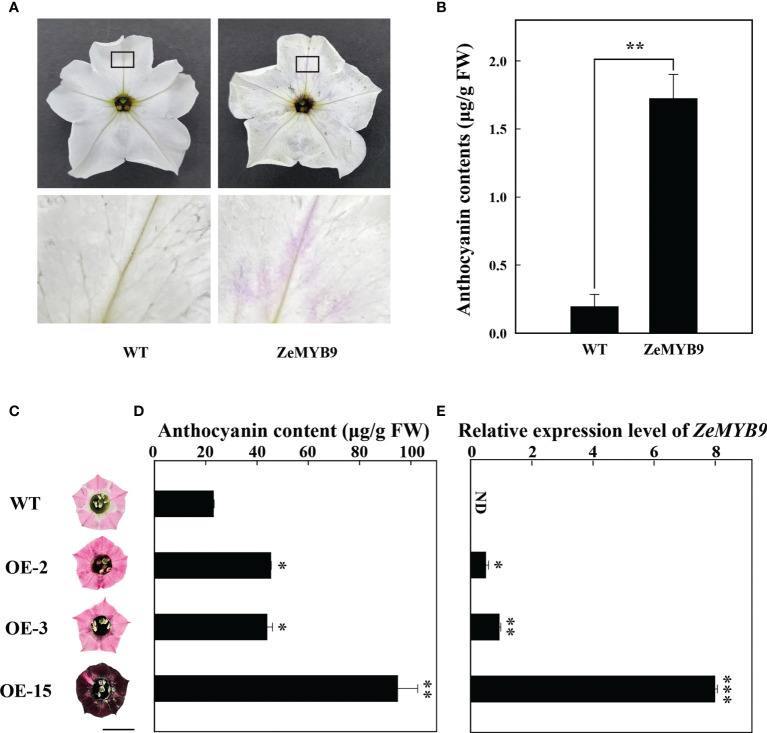
Overexpression of *ZeMYB9* in petunia and tobacco. **(A)** Phenotype of the entire (above) and the partial view (below) of petal limbs in wild type and *ZeMYB9*-overexpression flowers. **(B)** Total anthocyanin content in petal limbs of wild type and transiently transformed petunia. **(C)** Petal limb phenotype of wild-type and transgenic tobacco plants (OE-2, OE-3 and OE-15). The black line on the diagram showed a scale of 1cm. **(D)** Total anthocyanin contents in wild type and transgenic tobacco petal limbs. **(E)** Transcription levels of *ZeMYB9* in wild type and transgenic tobacco petal limbs. Data were showed as the mean ± SE from three biological replicates. T-test was used for statistical analyses compared with corresponding control (*P < 0.05, **P < 0.01, ***P < 0.001).

Transient transformation of petunia flowers and stable transformation of tobacco over-expressing *ZeMYB9* were used to characterize the function of *ZeMYB9*. Comparing with wild type, the petal limbs of transient petunia showed pink patches and obvious anthocyanin accumulation (**Figure 4A**). The content of anthocyanin in petunia petal limbs transiently expressing *ZeMYB9*, was about eight times higher than that in wild type (**Figure 4B**). As expected, in the transient expression petunia, the expression of nearly all of structural genes involved in anthocyanin synthesis was markedly upregulated (**Figure S2**).

**Figure 5:**
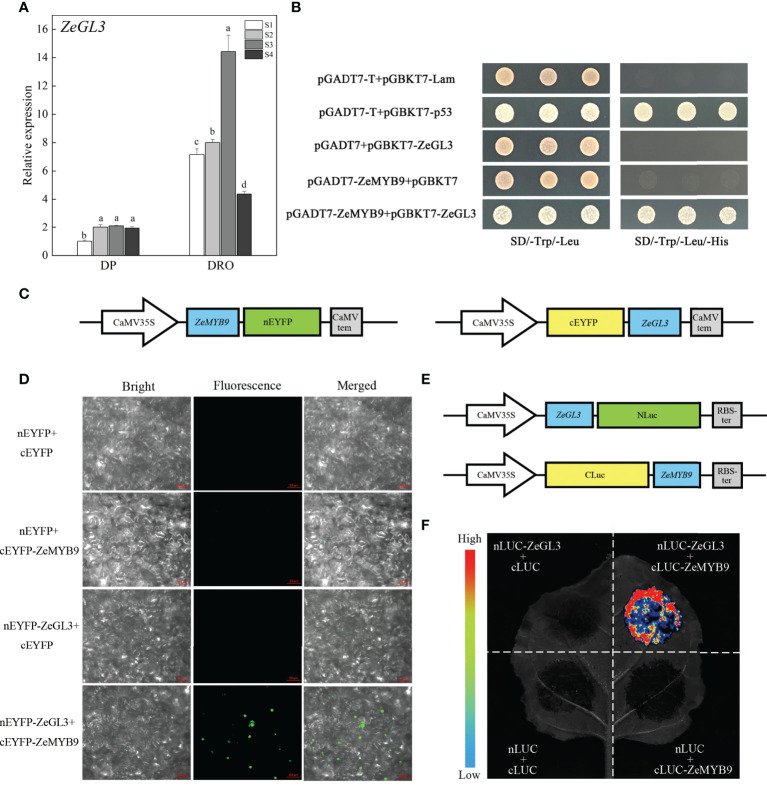
Expression analysis of ZeGL3 and interaction analysis of ZeMYB9 and ZeGL3. **(A)** Relative expression levels of ZeGL3 in the petals of DP and DRO at different developmental stages. ZeACT gene was used as an internal control for normalization, and three biological replicates were performed. Error bars indicate standard error (SE), and different lowercases indicate significant differences (P < 0.05). **(B)** Yeast two-hybrid assay for interaction relationship analysis between ZeMYB9 and ZeGL3. **(C)** Reporter plasmids construction diagrams for Bimolecular fluorescence complementation (BiFC) assays. **(D)** BiFC analysis between ZeMYB9 and ZeGL3 in N. benthamiana leaves. **(E)** Reporter plasmids construction diagrams for luciferase complementary assay (LCA). **(F)** ZeMYB9 could interact with ZeGL3 in LCA.

ZeMYB9 driven by 35S promoter was transformed into tobacco leaf discs by an *Agrobacterium*-mediated transformation. Several transgenic plants were transplanted to a climate chamber and used for subsequent phenotypic observation. Leaves of three transgenic lines (OE-2, OE-3, and OE-15) turned into dark red (**Figure S3A**), while obvious pigment accumulation showed in different floral tissues of the flowers of three transgenic plants, including petal limb (**Figure 5A**), tube, filament and anther (**Figure S3B**), compared with the wild type. Surprisingly, petal limb color of OE15 showed dark wine red (**Figure 4C**). Determination of anthocyanins from petal limb showed that the anthocyanin content in OE-2 and OE-3 was 2-fold higher than wild type, while that in OE-15 was 4-fold higher (**Figure 4D**), accompanied with the highest expression level of *ZeMYB9* (**Figure 4E**). Expression analysis showed that compared with the wild type, all the detected anthocyanin structural genes and *NtAN1a* were significant upregulated in petal limbs of OE-3 and OE-15, while only *NtCHS*, *NtF3H* and *NtF3′H* were significantly upregulated in OE2 (**Figure S4**). *NtAN1b* showed higher expression level only in OE-15 (**Figure S4**).

### ZeMYB9 could interact with ZeGL3 to activate the expression of *ZeF3’H*

bHLH proteins from IIIf subgroup are involved in the regulation of anthocyanin synthesis (Davies et al., 2012). A bHLH transcription factor belonging to IIIf subgroup was identified in *Z. elegans*, which was named ZeGL3 based on phylogenetic analysis that it was clustered into the clade of GL3 rather than clade of TT8 (**Figure S5A**). MYB-interaction region (MIR) and bHLH domain were present in ZeGL3 (**Figure S5B**). Similar to *ZeF3’H* and *ZeMYB9*, the expression level of *ZeGL3* was also greatly higher in DRO than that in DP (**Figure 5A**).

Yeast two-hybrid analysis showed that ZeGL3 and ZeMYB9 could physically interact, since yeast cells co-transformed with the positive control (pGBKT7-53+pGADT7-T) or pGADT7-ZeMYB9 with pGBKT7-ZeGL3, could grow on SD selection medium without Leu, Trp and His (**Figure 5B**). For BiFC assay, ZeGL3 and ZeMYB9 were individually fused with N-terminal and C-terminal fragment of enhanced yellow fluorescent protein (nEYFP and cEYFP) (**Figure 5C**). Then, different combinations were cotransformed into the same *N. benthamiana* leaf. No fluorescence was detected in negative control combinations, while strong fluorescence was observed in nucleus when coexpressing ZeMYB9-cEYFP and ZeGL3-nEYFP (**Figure 5D**). We also confirmed this result through luciferase complementary assay (LCA). ZeGL3 and ZeMYB9 were fused with NLuc and CLuc, respectively (**Figure 5E**). No LUC signal was detected in negative controls, while high LUC activity was present in the combination of ZeMYB9-CLuc and ZeGL3-NLuc (**Figure 5F**), which was consistent with the result of BiFC assay and indicated that ZeMYB9 could physically interact with ZeGL3.

**Figure 6:**
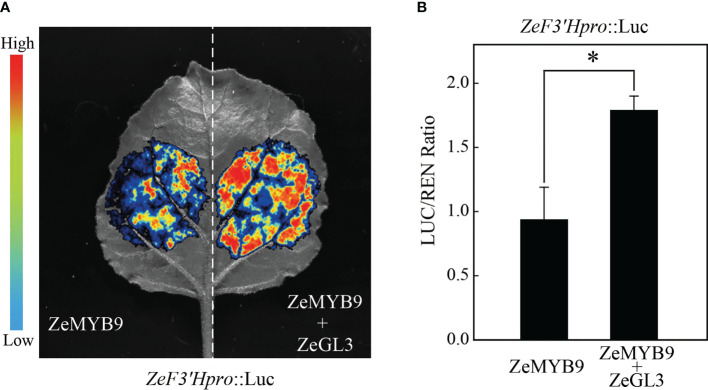
ZeMYB9 can form complexes with ZeGL3 to activate the expression of *ZeF3’H*. **(A)** Dual-luciferase assay in *N. benthamiana* leaves. **(B)** Ratio of LUC to REN activity. The data were the mean ± SE from three biological replicates. T-test was used for statistical analyses compared with corresponding control (*P < 0.05).

Luciferase assay showed that the relative activitiy of the *ZeF3’H* promoter was enhanced with the combination of ZeGL3 and ZeMYB9, compared to ZeMYB9 alone (**Figure 6**). These results revealed that ZeMYB9 could form a transcriptional complex with ZeGL3 to activate the expression of *ZeF3’H*.

### Co-expression of ZeMYB9 and ZeGL3 triggers the accumulation of anthocyanins in tobacco leave

**Figure 7:**
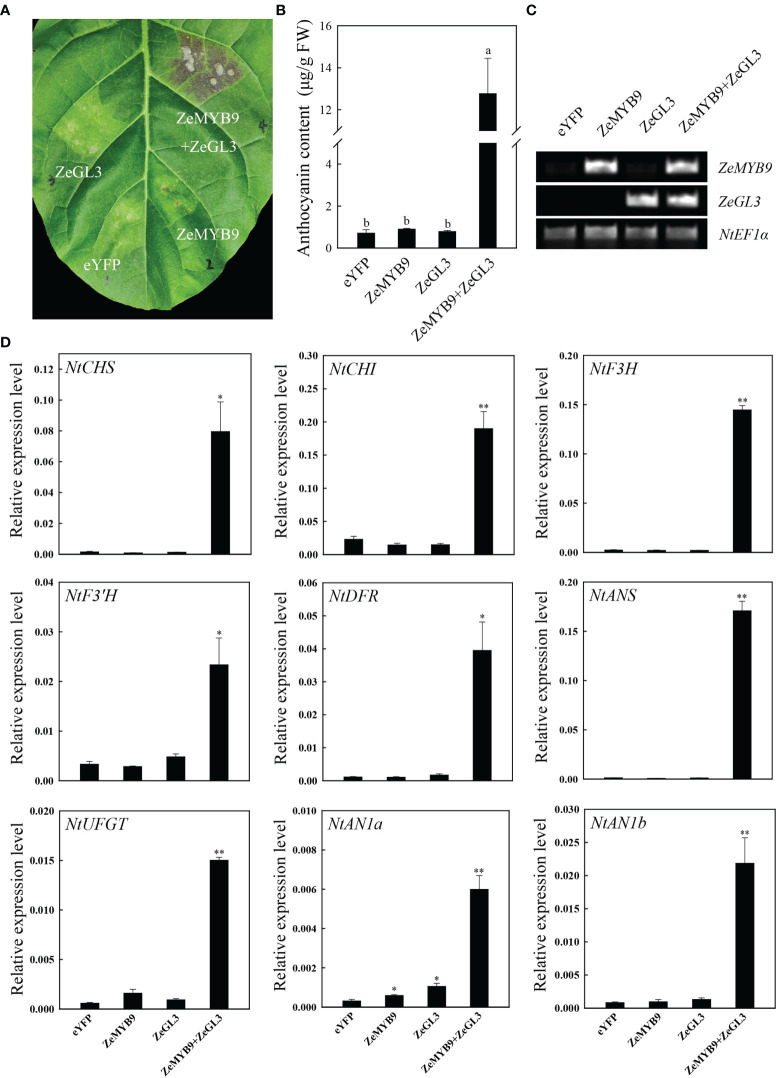
Co-expression of ZeMYB9 and ZeGL3 induces anthocyanin accumulation in tobacco leaves. **(A)** The tobacco leaves were photographed by camera and the location of gene injection was indicated on the graph. **(B)** Analysis of total anthocyanin content. Error bars indicate standard error (SE). And different lowercases indicate significant differences (*P* < 0.05). **(C)** Transcription levels of *ZeMYB9* and *ZeGL3* in the control and transient transformation of tobacco. *NtEF1α* was used as the reference gene. **(D)** Relative expression levels of the anthocyanin biosynthesis related structural genes and *bHLHs* (*AN1a* and *AN1b*). *NtEF1α* gene was used as an internal control for normalization, and three biological replicates were performed for qRT-PCR analysis. T-test was used for statistical analyses compared with corresponding control (*P < 0.05, **P < 0.01).

To further characterize the function of *ZeMYB9* and *ZeGL3* in anthocyanin synthesis, transient transformation in tobacco leaves was carried out. The tobacco leaves turned into red in the injection area after five days which co-expressed *ZeMYB9* and *ZeGL3*, while no red pigmentation was detected infiltrating with eYFP (empty vector), *ZeMYB9* alone or *ZeGL3* alone (**Figure 7A**). Co-expression of *ZeMYB9* and *ZeGL3* possessed the ability to produce a dramatic accumulation of anthocyanin, but *ZeMYB9* or *ZeGL3* alone could not promote anthocyanin biosynthesis in tobacco leaves (**Figure 7B**). In co-expression of *ZeMYB9* and *ZeGL3*, all structural genes and two *bHLH* genes were significantly upregulated compared to the control (**Figure 7D**).

## Discussion

The difference in the composition and content of anthocyanin provides many ornamental plants in a wide variety of colors. In flowers with pink, red or purple petal color, higher anthocyanin content is always accompanied with higher values of color parameters a* and C* and low value of color parameter L* (Zhang et al., 2014; Deng et al., 2019). Similarly, compared to DP cultviar, DRO higher anthocyanin accumulation exhibited higher a* and C* values, and lower L* value (**Figure S1B**). The synthesis of anthocyanin in petals is affected by the regulation of anthocyanin structural genes (Tanaka and Ohmiya, 2008). F3’H, a cytochrome P450 protein, belongs to CYP75B (Tanaka and Brugliera, 2013). *F3’H* gene could redirect the anthocyanin synthesis into cyanidin pathway and yield intense red color (Hoshino et al., 2003). The pink variety ‘Albert heijn’ of *Tulipa fosteriana* accumulated more cyanidins than the orange variety ‘SN09’, due to higher *F3’H* transcription leve (Yuan et al., 2014). When the expression levels of *F3’Hs* were downregulated, nearly only pelargonidin derivatives was accumulated in the pink flower varieties of *S. cruentus* (Jin et al., 2016). The pink and red flowers of *C. cyanus* had no cyanidins due to the open reading frame of *F3’H* were truncated and loss of a haem binding site (Deng et al., 2019). Similarly, we found the expression level of *ZeF3′H* in DRO was significantly higher than in DP (**Figure 2A**), accompanying with more accumulation of cyanidins in DRO (**Figure 1**).

Anthocyanin synthesis is controlled not only by structural genes, but also by regulatory genes. Currently, anthocyanin accumulation in many plants is regulated by R2R3-MYB transcription factors (Xu et al., 2015). R2R3-MYBs from *A. thaliana* could be divided into several subfamilies according to the conserved motifs, of which SG6 R2R3-MYBs act as activators of anthocyanin biosynthesis (Quattrocchio et al., 1999; Jian et al., 2019; Fang et al., 2021). ZeMYB9, a typical SG6 R2R3-MYB with conserved SG6 motif (**Figure 2C**) was proven to be a positive regulator of anthocyanin synthesis, because overexpression of this gene could greatly promote anthocyanin accumulation in petunia and tobacco flowers (**Figure 4**). SG6 R2R3-MYBs, such as MYB6 from *Populus tomentosa* and RH1 from *Medicago truncatula*, could directly bind to the MYB element on the promoter of the structural gene on the anthocyanin synthesis pathway to promote anthocyanin accumulation (Wang et al., 2019a; Wang et al., 2021). Similar to the expression pattern of *MaMybA* in grape hyacinth (*Muscari armeniacum*) (Chen et al., 2019), the expression of *ZeMYB9* increased first and then decreased with the development of flowers. What’s more, *ZeMYB9* showed a significant positive correlation with *ZeF3’H* expression level and specifically activated the promoter of *ZeF3’H* based on yeast one-hybrid and dual luciferase assays (**Figure 3**). It was suggested that ZeMYB9 can alter anthocyanin profile by regulating the expression of *ZeF3’H* in *Z. elegans*.

Numerous studies have identified that a conserved motif [D/E]Lx_2_[R/K]x_3_Lx_6_Lx_3_R in the R3 domain of R2R3-MYB could interact with bHLH of IIIf subgroup to form MYB-bHLH complex in all anthocyanin-accumulating plants (Feller et al., 2011). In *A. thaliana*, IIIf bHLH transcription factors are mainly divided into TT8 and GL3 clades. AtTT8 can interact with MYB and WD40 to regulate the synthesis of anthocyanin and proanthocyanidins (Baudry et al., 2004; Gonzalez et al., 2008). AtGL3 and AtEGL3 can also coordinate with MYB transcription factors to regulate the synthesis of anthocyanin but not proanthocyanidins (Payne et al., 2000; Morohashi et al., 2007). *PbbHLH2* from *Pyrus bretschneideri*, interacts with MYB through the N-terminal MIR domain to promote anthocyanin synthesis (Li et al., 2021). Evolutionary analysis showed that ZeGL3 belonged to the GL3 clade and there was a MIR domain region present in its N-terminal (**Figure S5**), suggesting that this protein may possess positive regulation on anthocyanin synthesis. In our study, yeast two-hybrid, LCA and BiFC assays illustrated the interaction between ZeMYB9 and ZeGL3 proteins (**Figure 5**). AcMYB123 and AcbHLH42 in *Actinidia chinensis* cv. Hongyang could interact to induce anthocyanin accumulation in tobacco leaves and activate the expression of *AcF3GT1* and *AcANS* promoter (Wang et al., 2019b). Similar positive function of MYB-bHLH complex on the expression of anthocyanin structural genes was well conserved in many plants, such as *Anthurium andraeanum* (Li et al., 2019a), *P. qiui* (Liu et al., 2022), *Medicago truncatula* (Li et al., 2016), etc. In our study, the interaction of ZeMYB9 and ZeGL3 proteins could enhance the activation of *ZeF3’H* expression (**Figure 6**) and trigger anthocyanin accumulation in tobacco leaves (**Figure 7**).

**Figure 8:**
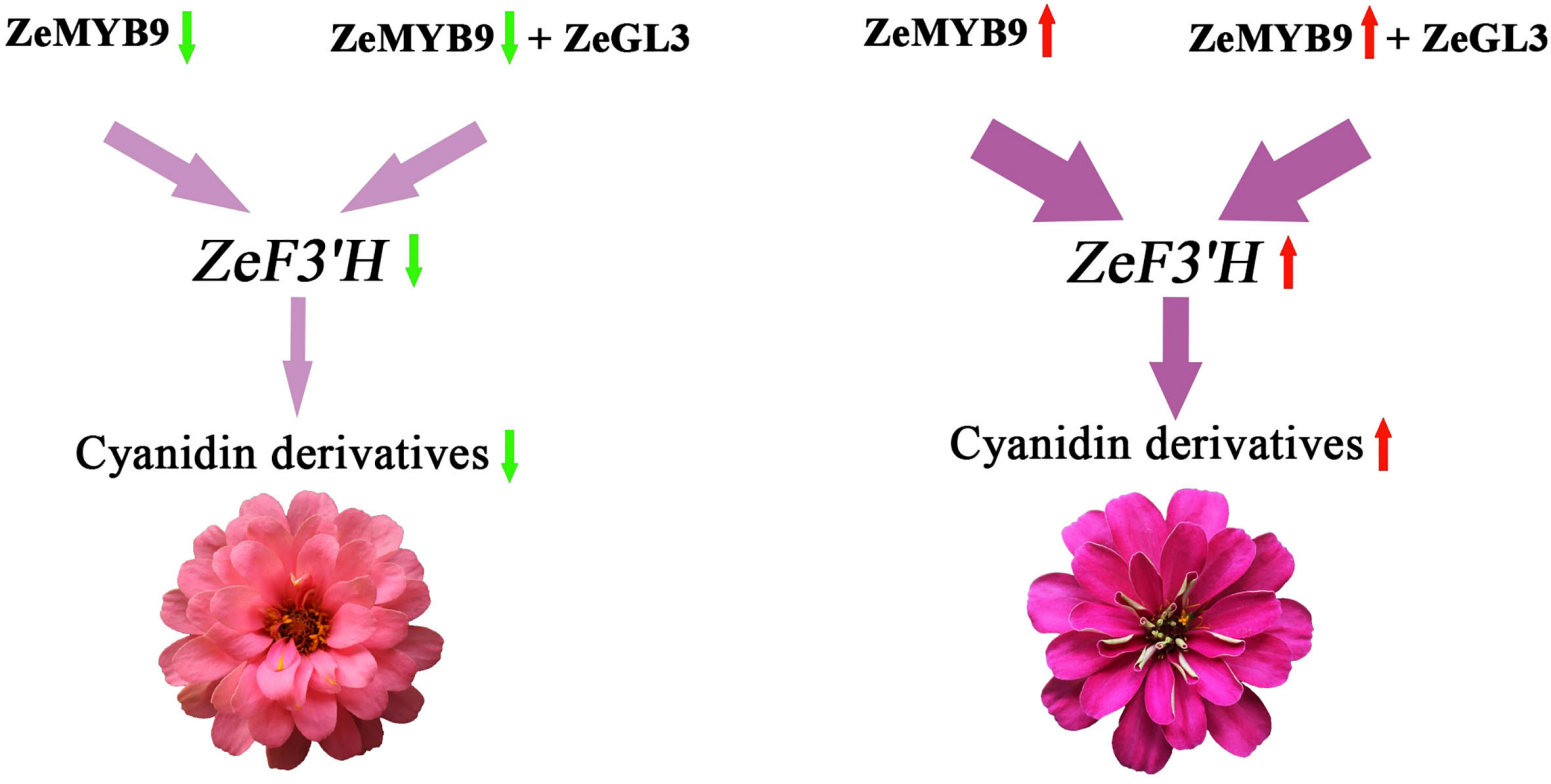
Diagram of regulatory pattern of *ZeMYB9* in *Z. elagans*.

On the basis of the above results, we propose a regulatory network of anthocyanin biosynthesis in *Z. elegans*, which was controlled by *ZeMYB9* (**Figure 8**). ZeMYB9 could directly or in the form of ZeMYB9-ZeGL3 complex bind to the promoter of *ZeF3’H*. Higher expression of *ZeMYB9* increased the expression of *ZeF3’H*, thereby anthocyanin synthesis was more directed to the DHQ pathway and eventually to the synthesis of cyanidins in DRO. Meanwhile, the expression of *ZeMYB9* in DP was relatively lower, resulting in downregulated expression of *ZeF3’H*, so the synthesis of anthocyanin mainly led to the accumulation of pelargonidins. These results would significantly expand the current understanding of transcriptional regulation of flower color in *Z. elegans*, and also provide theoretical basis for flower color breeding in Compositae flowers.

## Conclusion

Cyanidins were the main pigments that affected the color of DP and DRO petals. We found a positive MYB regulator of the S6 subfamily, ZeMYB9, which was positively correlated with *ZeF3’H* expression and higher expression of *ZeMYB9* and *ZeF3’H* was detected in the DRO cultivar with higher cyanidin content. ZeMYB9 could significantly activate the expression of *ZeF3’H* promoter and promoted anthocyanin accumulation in petunia and tobacco corolla. ZeMYB9 was also shown to interact with a IIIf subfamily of bhLH transcription factor ZeGL3 to promote *ZeF3’H* promoter expression and induce anthocyanin synthesis in tobacco leaves. This study revealed the regulation mechanism of color difference of different *Z. elagans* cultivars, which provides useful resources for future studies on the molecular breeding of flowers color in Compositae flowers.

## Materials and methods

### Plant materials and growth conditions

*Z. elegans* ‘Dreamland Pink’ (DP) with pink petal color and ‘Dreamland Rose’ (DRO) with purple petal color, were chosen as materials (**Figure 1A**). These plant materials were grown in climatic chamber with a relative humidity of 60% and long-day conditions (16 h/8 h light/dark, 25°C/18°C). Flower opening process of *Z. elegans* could be divided into four developmental stages (Qian et al., 2021): S1 was defined as when the ray florets were acicular and had barely outgrown the bract; S2 was defined as when the ray florets clearly outgrew and began to colored, the bract and the angle between the ray florets and the stem was equal to or less than 45°; S3 was defined as when petals of the ray florets were slightly flattened and completed coloring, the angle between the ray florets and the stem was about 90°; S4 was defined as when petals of the ray florets were flattened. The ray florets at the four developmental stages of DP and DRO were separated, immediately frozen in liquid nitrogen and stored at -80°C until use. Tobacco (*Nicotiana tabacum* ‘NC89’ or *N. benthamiana*) and *Petunia hybrida* ‘Mitchell’ used for subsequent experiments were also cultivated in long-day climatic chamber (16 h/8 h light/dark, 25°C/18°C).

### Color parameters and total flavonoids measurement

Color parameters of the middle part of ray florets in DP and DRO at S4 were determined by the color reader CR-10 (Konica Minolta Optics, Inc., Sakai, Japan). Lightness (L*) and two chromatic components a* and b* were analyzed using the International Commission on Illumination (CIE) L*a*b*system (Gonnet, 1993). Chroma (C*) and hue angle (*h°*) were calculated by L*, a* and b* values: C*=(a*^2^+b*^2^)^1/2^, *h°*=arctan (b*/a*) (McGuire, 1992).

The total flavonoids contents in the ray florets of DP and DRO at S4 were quantified as described by Sakata (Sakata et al., 1995) and the content was calculated by a standard curve drawn with rutin as the standard substance: Total flavonoids=0.0078*A_405_ (R^2 =^ 0.993).

### Extraction and component analysis of anthocyanins

0.2 g fresh ray florets of DP and DRO at S4 were weighed and ground into powder using liquid nitrogen. Then, they were incubated in 2 mL extracting solution containing 2% formic acid and 70% methyl alcohol overnight at 4°C (Qian et al., 2021). The combined supernatants were filtered through 0.22 μm micropore filter for further analysis by UPLC. The UPLC (Waters Acquity H-Class) was equipped with chromatographic column using Acquity UPLC^®^ HSS T3 (Waters, 1.8 μm, 2.1×100 mm) and the column temperature was 30°C. The analytical column was eluted with mobile phase A (formic acid: water, 1:99, v/v) and mobile phase B (acetonitrile) at a rate of 0.4 mL min^−1^. The gradient elution condition was: 0-15 min, 95%-5% A, 5%-95% B; 15-16 min, 5% A, 95% B; 16.0-16.1 min, 5%-95% A, 95%-5% B; 16.1-19.0 min, 95% A, 5% B. The UV detection wavelength was set at 530 nm. Quantitative analysis of each component was conducted by external standard method using cyanidin-3-*O*-rutinoside as a standard.

### Gene sequence and phylogenetic analysis

Gene sequences of *ZeMYB9* and *ZeGL3* were identified from petal transcriptome of *Z. elegans*. The amino acid sequences of *ZeMYB9* and *ZeGL3* were subjected to multiple sequence alignment by DANMAN and phylogenetic tree analysis by MEGA 6 (Tamura et al., 2011) with the neighbor-joining method (1000 bootstrap replicates). The anthocyanidin-related MYB and bHLH proteins in *Arabidopsis thaliana* and other species were obtained from the TAIR (https://www.arabidopsis.org/) and NCBI (https://www.ncbi.nlm.nih.gov/) databases.

### RNA extraction and cDNA synthesis

Total RNA was extracted using the FastPure Plant Total RNA Isolation Kit (Vazyme, Nanjing, China). The cDNA was synthesized from the PrimeScript™ RT Master Mix (Takara, Dalian, China).

### qRT-PCR analysis

The expression of *ZeMYB9*, *ZeF3’H* and *ZeGL3* at four developmental stages in two cultivars was analyzed. The RT-PCR reactions were performed using AceQ Universal SYBR qPCR Master Mix (Vazyme, China) on LightCycler^®^ 480 II (Roche Diagnostics, Germany). Reactions were run with three biological replicates and the amplification program consisted of one cycle of 95°C for 5 min followed by 40 cycles of 95°C for 10 s and 60°C for 30 s with melt curve program. The *ZeACT* gene was used to normalize the samples. Relative expression of the target genes was calculated by 2^−ΔΔCt^ (Livak and Schmittgen, 2001). qRT-PCR data were presented here as the mean ± SE of three biological replicates. All primer sequences were available in **Table S1**.

### *ZeF3’H* promoter isolation and analysis

*ZeF3’H* gene was predicted as the key anthocyanin biosynthetic gene determining the synthesis of cyanidins in *Z. elegans* (Qian et al., 2021). The promoter of *ZeF3’H* was isolated with genomic walking FPNI-PCR (Wang et al., 2011) from genomic DNA of young leaves extracted with CTAB method (Murray and Thompson, 1980). Primers used in FPNI-PCR were showed in **Table S2**. And the *cis-*elements of *ZeF3’H* promoter sequence were predicted by PlantCARE (http://bioinformatics.psb.ugent.be/webtools/plantcare/html/).

### Yeast one-hybrid assay

The promoter sequence of *ZeF3’H* (ZeF3’H-Pro) was amplified with primers in **Table S2** and inserted into the pAbAi vector, and the CDS of *ZeMYB9* was cloned into the pGADT7 vector with the primers listed in **Table S3**. pAbAi-ZeF3’H-Pro was transformed into Y1H Gold according to the protocol of Yeastmaker™ Yeast Transformation System 2 User Manual, and the lowest inhibitory concentration of aureobasidin A (AbA) for the bait strain was tested on SD/-Ura media. The prey and was transformed into yeast cells containing recombinant pAbAi vector and then incubated on SD/-Leu medium at 30°C for 3 days. The interactions between pGADT7-ZeMYB9 and pAbAi-ZeF3’H-Pro were detected on SD/-Leu medium with 100 ng mL^-1^ AbA at 30°C for 4 days.

### Dual luciferase assay

The full-length fragment of the *ZeF3’H* promoter was cloned into pGreenII 0800-LUC vector and the CDS of *ZeMYB9* and *ZeGL3* were inserted into pCNHP-eYFP vector, respectively. The recombinant plasmids and empty vector were transformed into *Agrobacterium* strain GV3101 or GV3101 (pSoup). The *Agrobacterium* strain bacteria that carried transcription factor or the promoter were resuspended in infiltration medium (10 mM MgCl_2_ and 200 μM acetosyringone) and mixed when OD_600_ reached to 0.8. Then the mixtures were injected into tobacco leaves without needle. After three days, the transformed tobacco leaves were sprayed with D-luciferin sodium salt (Coolaber, Beijing, China) and then observed using Fuison-FX7 multi-imaging apparatus (Vilber, France). Enzyme activities of Firefly luciferase (LUC) and Renilla luciferase (REN) were tested using Dual Luciferase Reporter Assay Kit (Vazyme, China).

### Transient transformation of petunia flowers

In order to verify the function of *ZeMYB9* in the regulation of anthocyanin biosynthesis, transient overexpression of *ZeMYB9* in petunia flowers were infiltrated by vacuum infiltration method. Day 1 petunia flowers were respectively submerged in infiltration containing *Agrobacterium* (OD_600 =_ 1.0) harboring pCNHP-eYFP-*ZeMYB9* or empty vector and subjected to vacuum condition of -13 psi. After releasing the vacuum, the petals were cultured in 2 mL tubes containing 3% sucrose for five days. And then, the petal limbs were observed and sampled for anthocyanin measurement and gene expression analysis. The expression level of *ZeMYB9* and anthocyanin related genes in petunia petals were analyzed by using qRT-PCR. The qRT-PCR primers for petunia anthocyanin-related structural genes and *PhEF1α* were obtained from the previous study (Zhang et al., 2021).

### Stable tobacco transformation

The CDS of *ZeMYB9* was inserted into pCNH-Flag vector, and the recombinant plasmid was transformed into *Agrobacterium* strain GV3101 for transformation. Then the *Agrobacterium* carrying pCNH-Flag-*ZeMYB9* plasmid inflected *N. tabacum* ‘NC89’ leaf disks according to the protocol described by Li’s methods (Li et al., 2019b). The transformed tobacco plants were screened using 10 mg L^−1^ hygromycin antibiotic. The petal limbs of transgenic plants and wild-type tobacco were used for analyzing anthocyanin contents and gene expression levels. The expression level of *ZeMYB9* and anthocyanin related genes in tobacco petal limbs was analyzed by qRT-PCR. The qRT-PCR primers for tobacco anthocyanin-related structural genes and *NtEF1α* were obtained from the previous study (Li et al., 2019b).

### Yeast two-hybrid assay

The full-length coding sequences of *ZeMYB9* and *ZeGL3* were respectively introduced into pGADT7 and pGBKT7 vectors. Various combinations of BD and AD vectors were cotransformed into the yeast strain Y2H Gold and grown on SD/-Leu-Trp medium at 30°C for 3-4 days. The clones were subsequently grown on SD/-His-Leu-Trp medium at 30°C for 4 days to test the interaction between ZeMYB9 and ZeGL3.

### BiFC assay and LCA

Bimolecular fluorescence complementation (BiFC) and luciferase complementary assay (LCA) were used to analyze the interaction between ZeMYB9 and ZeGL3 proteins. Full-length sequences of ZeMYB9 and ZebHLH1 were cloned into the vector pCNHP-cEYFP and pCNHP-nEYFP, respectively. The recombinant plasmids were respectively transformed into *Agrobacterium* strain GV3101 and then different combinations of *Agrobacterium* strains were co-transformed into four-week tobacco leaves with needleless syringes which resuspended in infiltration medium (10 mM MgCl_2_ and 200 μM acetosyringone) to each OD_600_ of 0.8. After 48 h of the injection, EYFP fluorescence was detected using Fluorescence microscope of Axio Imager A2 (ZEISS, Germany).

Full-length sequences of *ZeMYB9* and *ZeGL3* were inserted into the pCAMBIAI1300-Cluc and pCAMBIAI1300-Nluc plasmids, respectively. All combinations of *Agrobacterium* strains cell containing recombinant plasmids were infiltrated into tobacco leaves. The infiltrated tobacco leaves were sprayed with D-luciferin sodium salt (Coolaber, Beijing, China) and detected after three days using Fuison-FX7 multi-imaging apparatus (Vilber, France).

### Transient transformation of tobacco

Transient co-expression of *ZeMYB9* and *ZeGL3* in *N. tabacum* ‘NC89’ leaves was conducted based on a modified method (Sparkes et al., 2006). *Agrobacterium* containing pCNHP-eYFP-*ZeMYB9*, pCNHP-eYFP*-ZeGL3* or the mixture were infiltrated into tobacco leaves and the plants were cultured in phytotron under normal condition for five days. Transcription of *ZeMYB9* or *ZeGL3* was analyzed by RT-PCR, while anthocyanin biosynthesis-related structural genes and *bHLHs* (*AN1a* and *AN1b*) were analyzed by qRT-PCR. The *NtEF1α* gene was used to normalize the target genes.

### Total anthocyanin determination

Anthocyanin contents were measured using the procedure of previous study (Stevenson and Harrington, 2009). The injected tobacco leaves or petunia flowers were ground to a powder in liquid nitrogen and then extracted in 2 mL of acidified methanol (1% HCl) overnight at 4°C and in the dark. Chloroform was added to remove chlorophyll that tobacco leaves to prevent interference with absorbance, and anthocyanin were measured at 530 and 657 nm. Relative anthocyanin content was calculated on a formula: (A_530_ – 0.25 * A_657_)/mg FW.

### Statistical analysis

One-way analysis of variance (ANOVA) or T-test was considered appropriate to determine significant differences between pairwise comparisons in the analyses of color parameters, pigment contents or gene expression level with SPSS software version 20.0 (SPSS Inc., Chicago, IL, USA). Photographs were created using SigmaPlot software version 14.0 (Systat Software, San Jose, CA, USA), Adobe Photoshop CC 2019 and Adobe Illustrator CC 2019 (Adobe Systems, Inc., San Jose, CA, USA).

## Data availability statement

The sequence data presented in the study are deposited in the NCBI GenBank database, accession numbers MT815440, ON959236 and ON959237 for *ZeF3′H*, *ZeMYB9* and *ZeGL3*, respectively.

## Author contributions

CZ and JF designed and supervised this study. JQ and LJ performed the experiments and analyzed the data. HQ, JC, ZW and MX assisted with doing the experiments. JQ, LJ and CZ wrote the article. All authors contributed to the article and approved the submitted version.

## Funding

This research was supported by National College Students Innovation and Entrepreneurship Training Program (No. 202010341022), College Students Research Training Program of Zhejiang Agriculture and Forestry University (No. 2021KX0069 and S202210341185), and the Open Fund of Zhejiang Provincial Key Laboratory of Germplasm Innovation and Utilization for Garden Plants (K202105).

## Acknowledgments

We are grateful to Dr. Xing-Qi Huang for the gift of plasmids pCNHP-eYFP, pCNHP-cEYFP, pCNHP-nEYFP and pCNH-Flag.

## Conflict of interest

The authors declare that the research was conducted in the absence of any commercial or financial relationships that could be construed as a potential conflict of interest.

## Publisher’s note

All claims expressed in this article are solely those of the authors and do not necessarily represent those of their affiliated organizations, or those of the publisher, the editors and the reviewers. Any product that may be evaluated in this article, or claim that may be made by its manufacturer, is not guaranteed or endorsed by the publisher.

## Supplementary material

Supplementary Figure 1 Color parameters and total flavonoid content analysis of DP and DRO. **(A)** Total flavonoid content in DP and DRO. **(B)** The flower color parameter values of DP and DRO. *L**, lightness; *a**, redness; *b**, yellowness; *C**, chroma; *h°*, hue angle. Three biological replicates were performed for content analysis. T-test was used for statistical analyses compared with corresponding control (**P* < 0.05, ***P* < 0.01).

Supplementary Figure 2 Relative expression levels of *ZeMYB9* and structural genes in the anthocyanin biosynthesis pathway in the petal limbs of wild type and transgenic petunia. *PhEF1α* gene was used as an internal control for normalization, and three biological replicates were performed. Error bars indicated standard error (SE). T-test was used for statistical analyses compared with wild type (**P* < 0.05, ***P* < 0.01).

Supplementary Figure 3 Phenotypic observation of wild type and three transgenic lines (OE-2, OE-3 and OE-15). **(A)** Phenotypic observations of the entire plants. **(B)** Phenotypic observations of tubes, calyxs, anthers, filaments, and ovaries. The black line on the diagram showed a scale of 1cm.

Supplementary Figure 4 Relative expression levels of *ZeMYB9* and structural genes in the anthocyanin biosynthesis pathway in the petal limbs of wild type and transgenic tobacco. *NtEF1α* gene was used as an internal control for normalization, and three biological replicates were performed. Error bars indicated standard error (SE). T-test was used for statistical analyses compared with corresponding control (*P < 0.05, **P < 0.01, ***P < 0.001).

Supplementary Figure 5 Analysis of ZeGL3 from IIIf subgroup in *Zinnia elegans*. **(A)** Phylogenetic tree of different GL3 and TT8 proteins participating in anthocyanin biosynthesis in other plants. **(B)** Multiple alignment of the amino acid sequences of bHLH IIIf subgroup proteins from different plants. The black line at the top indicated the conserved MYB-interaction region (MIR) at the N-terminal. The alternating red and black lines indicated a bHLH domain at the C-terminal. The GenBank accession numbers of these bHLH proteins were as follows: the GL3 clade as Arabidopsis thaliana AtMYB75|PAP1 (AT1G56650.1), *Malus domestica* MdbHLH33 (ABB84474.1), *Fragaria ananass* FabHLH33 (AFL02465.1), *Vitis vinifera* VvMYCA1 (ABM92333.3), *Arabidopsis thaliana* AtGL3 (NP_001333705.1), *Antirrhinum majus* AmDEL (AAA33663.1); the TT8 clade as *Chrysanthemum morifolium* CmbHLH2 (ALR72603.1), *A. thaliana* AtTT8 (CAC14865.1), *Nicotiana tabacum* NtAn1a (AEE99257.1).

Supplementary Table 1 Primer sequences for qRT-PCR.

Supplementary Table 2 Primer sequences for promoter cloning.

Supplementary Table 3 Primer sequences for vector construction.
