# Supplementary figures and images for: ZeMYB9 regulates cyanidin synthesis by activating the expression of flavonoid 3′-hydroxylase gene in Zinnia elegans
Source: Front Plant Sci. 2022 Oct 18;13:981086. doi: 10.3389/fpls.2022.981086 (PMC9623174; doi:10.3389/fpls.2022.981086)

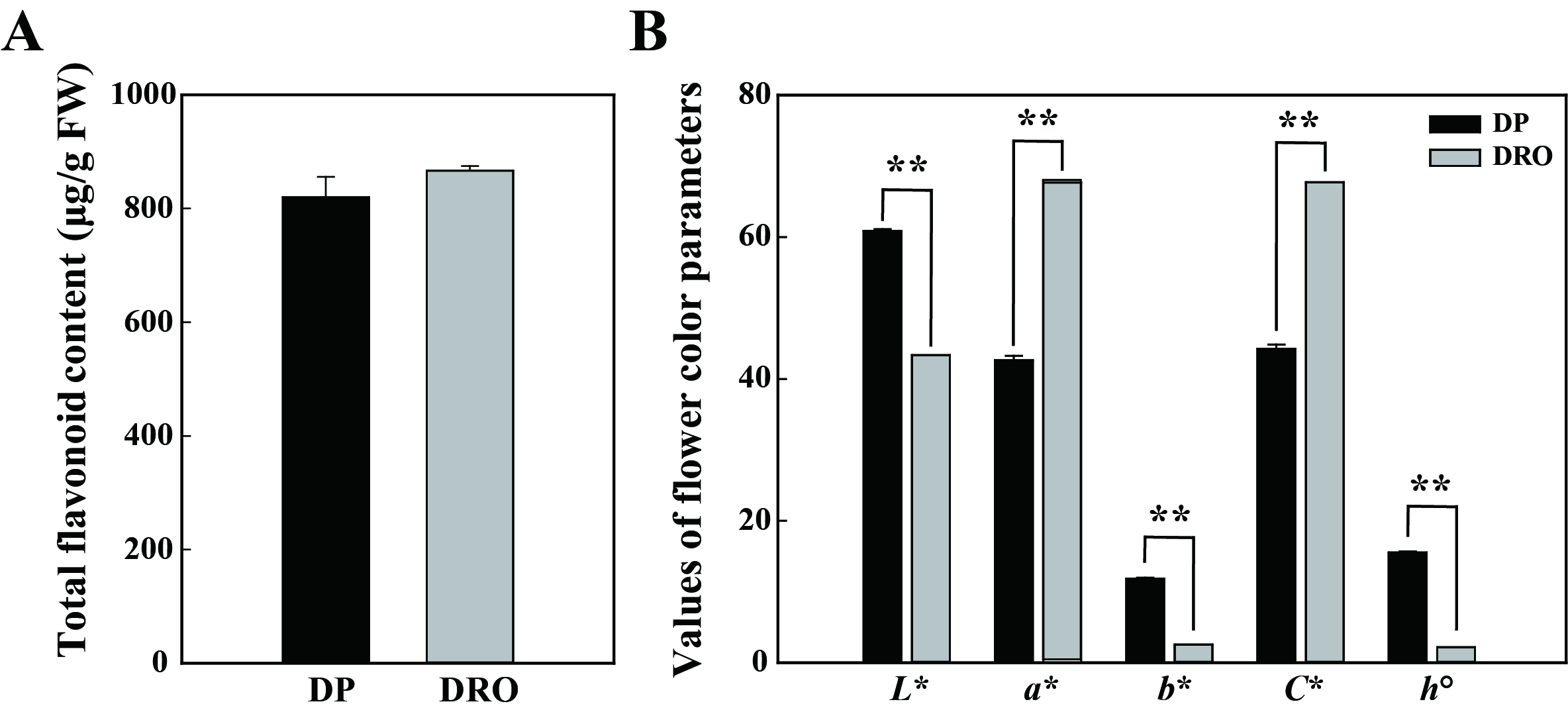

Supplement: Supplementary Figure 1 — Color parameters and total flavonoid content analysis of DP and DRO. (A) Total flavonoid content in DP and DRO. (B) The flower color parameter values of DP and DRO. L*, lightness; a*, redness; b*, yellowness; C*, chroma; h°, hue angle. Three biological replicates were performed for content analysis. T-test was used for statistical analyses compared with corresponding control (*P < 0.05, **P < 0.01). [file Image_1.tif]

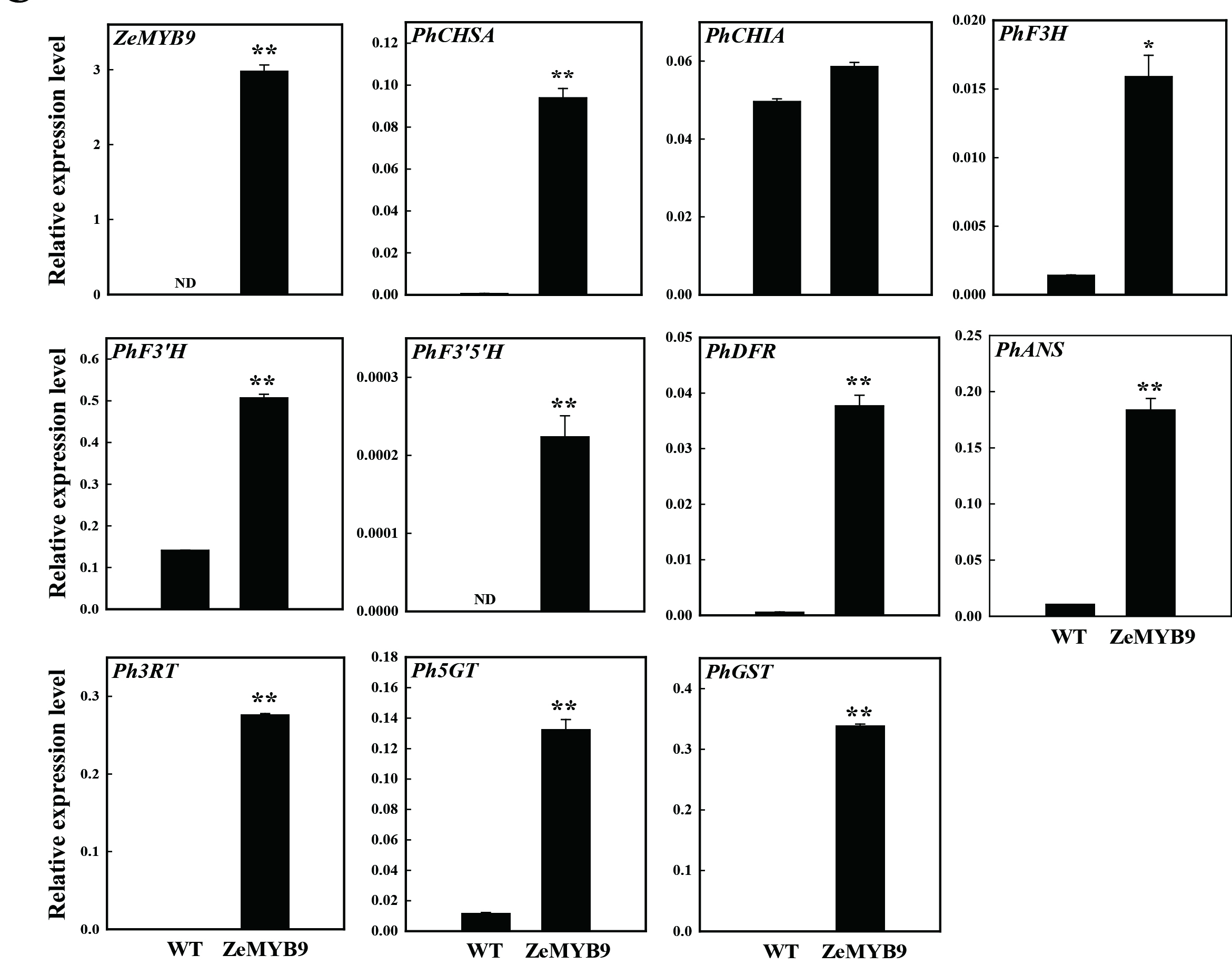

Supplement: Supplementary Figure 2 — Relative expression levels of ZeMYB9 and structural genes in the anthocyanin biosynthesis pathway in the petal limbs of wild type and transgenic petunia. PhEF1α gene was used as an internal control for normalization, and three biological replicates were performed. Error bars indicated standard error (SE). T-test was used for statistical analyses compared with wild type (*P < 0.05, **P < 0.01). [file Image_2.tif]

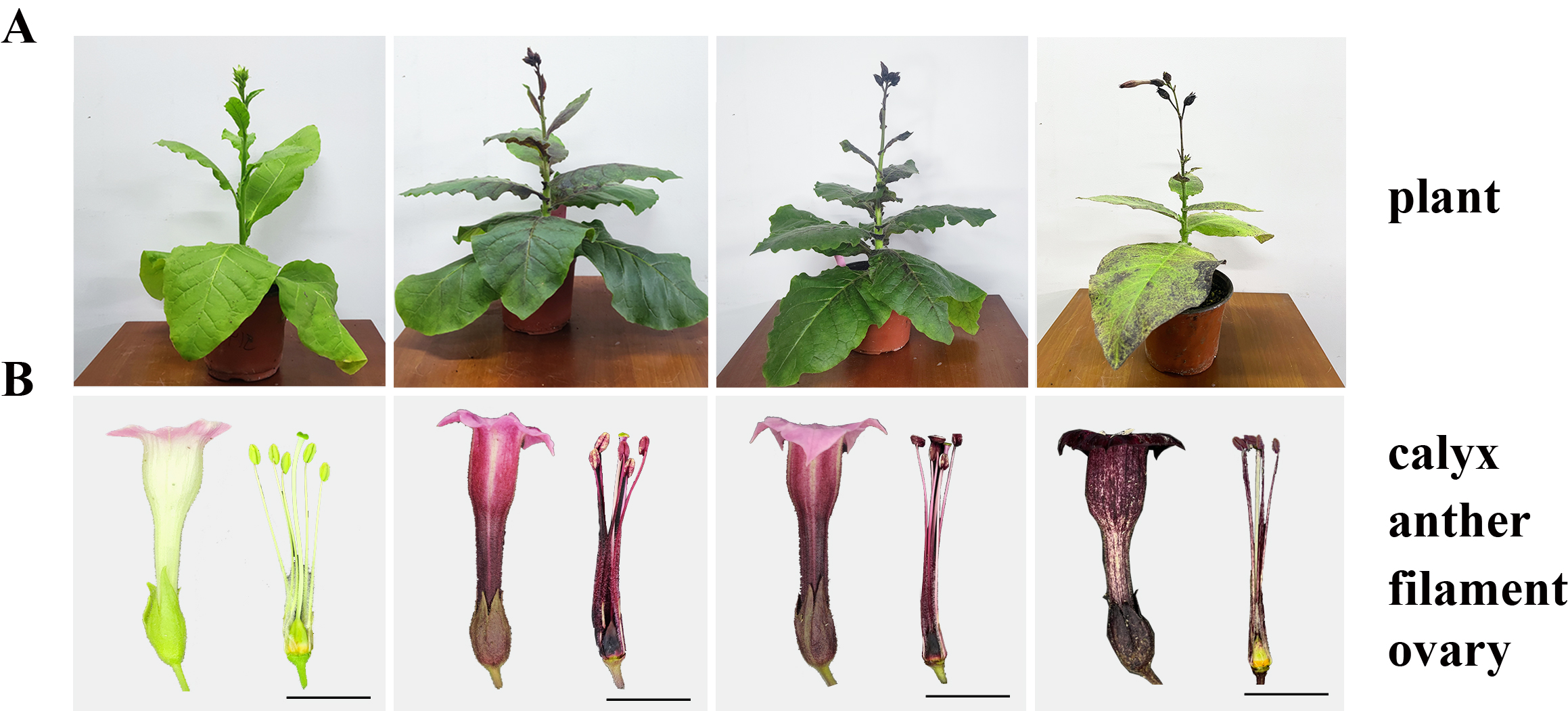

Supplement: Supplementary Figure 3 — Phenotypic observation of wild type and three transgenic lines (OE-2, OE-3 and OE-15). (A) Phenotypic observations of the entire plants. (B) Phenotypic observations of tubes, calyxs, anthers, filaments, and ovaries. The black line on the diagram showed a scale of 1cm. [file Image_3.jpeg]

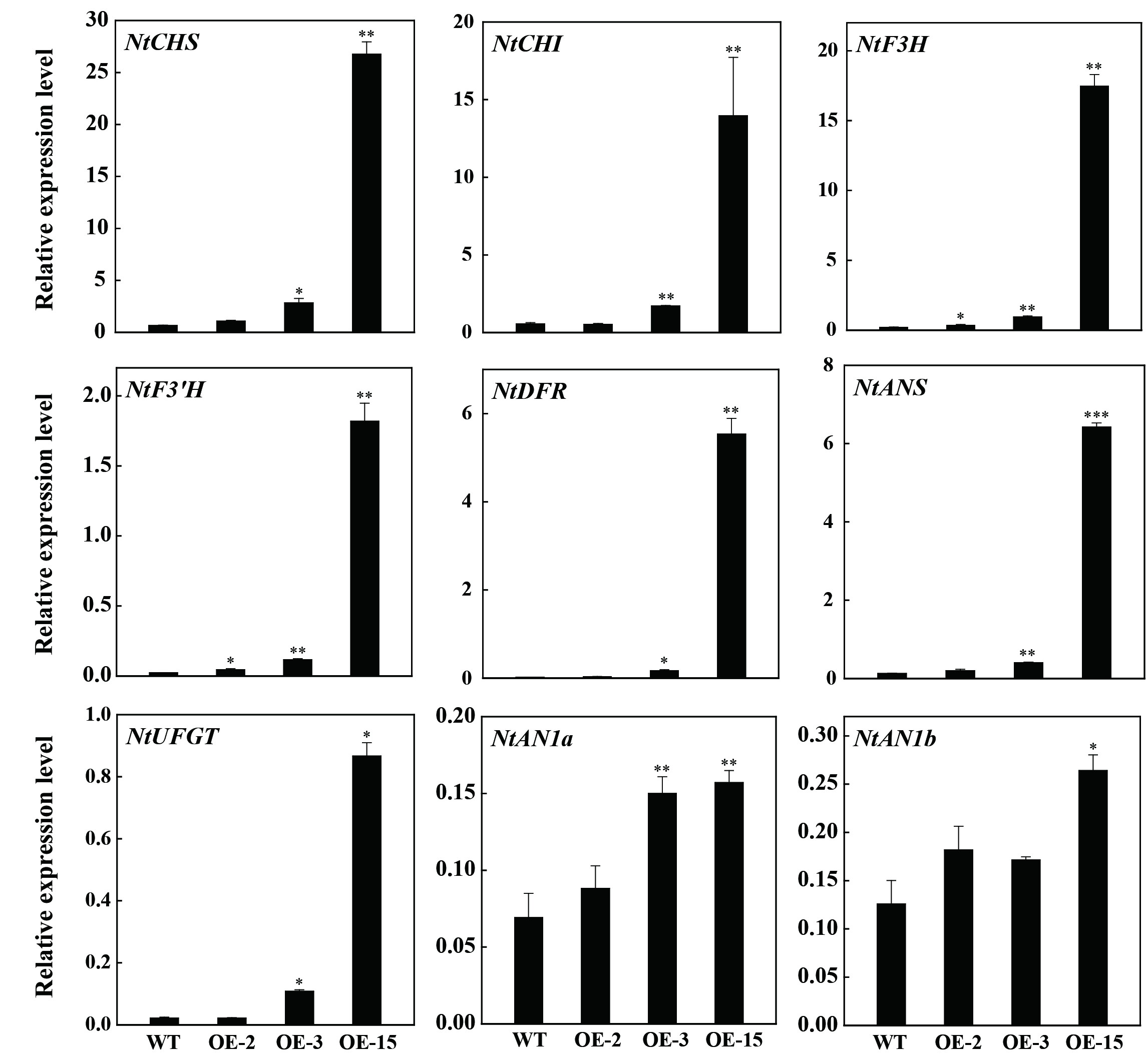

Supplement: Supplementary Figure 4 — Relative expression levels of ZeMYB9 and structural genes in the anthocyanin biosynthesis pathway in the petal limbs of wild type and transgenic tobacco. NtEF1α gene was used as an internal control for normalization, and three biological replicates were performed. Error bars indicated standard error (SE). T-test was used for statistical analyses compared with corresponding control (*P < 0.05, **P < 0.01, ***P < 0.001). [file Image_4.tif]

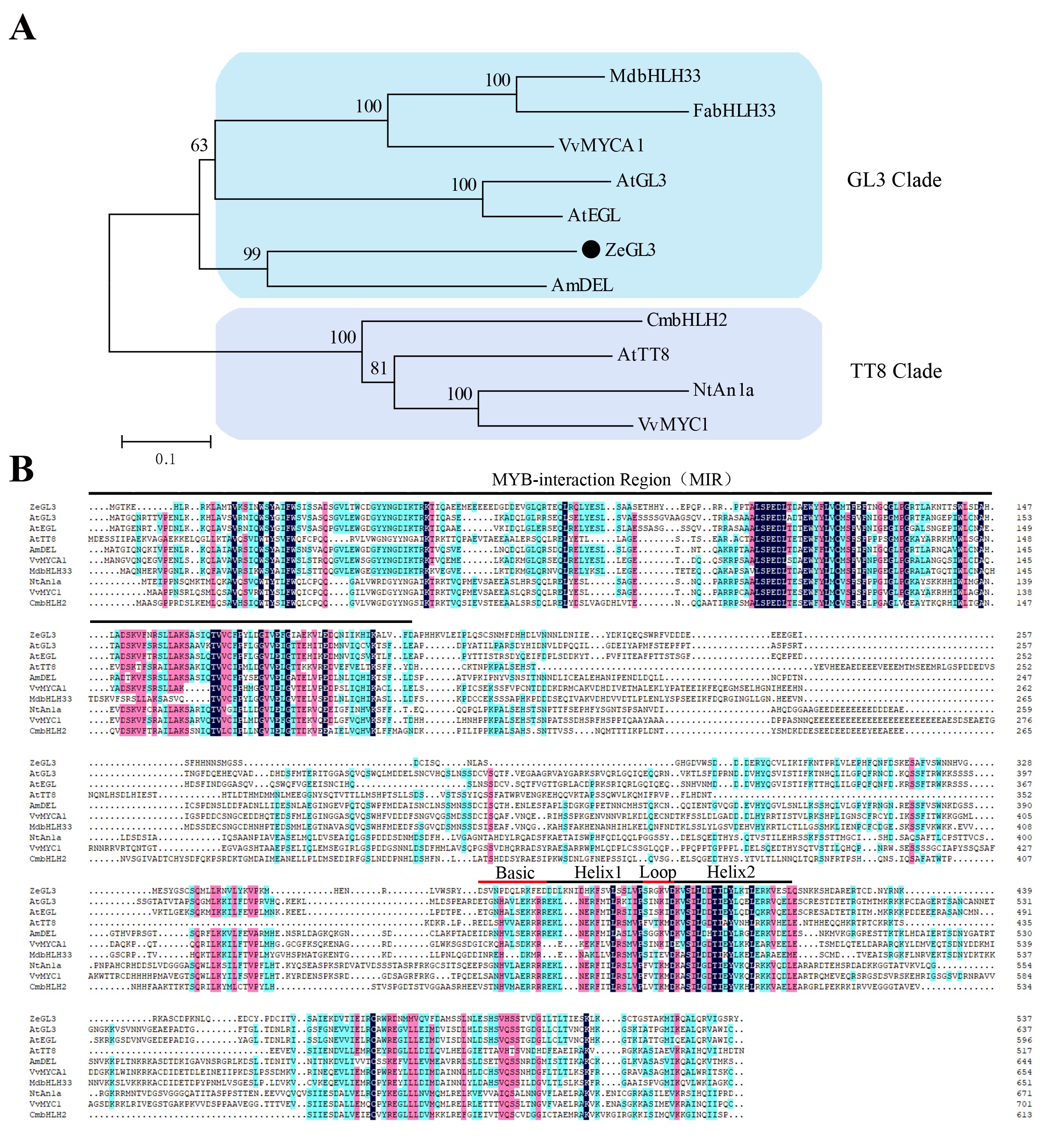

Supplement: Supplementary Figure 5 — Analysis of ZeGL3 from IIIf subgroup in Zinnia elegans. (A) Phylogenetic tree of different GL3 and TT8 proteins participating in anthocyanin biosynthesis in other plants. (B) Multiple alignment of the amino acid sequences of bHLH IIIf subgroup proteins from different plants. The black line at the top indicated the conserved MYB-interaction region (MIR) at the N-terminal. The alternating red and black lines indicated a bHLH domain at the C-terminal. The GenBank accession numbers of these bHLH proteins were as follows: the GL3 clade as Arabidopsis thaliana AtMYB75|PAP1 (AT1G56650.1), Malus domestica MdbHLH33 (ABB84474.1), Fragaria ananass FabHLH33 (AFL02465.1), Vitis vinifera VvMYCA1 (ABM92333.3), Arabidopsis thaliana AtGL3 (NP_001333705.1), Antirrhinum majus AmDEL (AAA33663.1); the TT8 clade as Chrysanthemum morifolium CmbHLH2 (ALR72603.1), A. thaliana AtTT8 (CAC14865.1), Nicotiana tabacum NtAn1a (AEE99257.1). [file Image_5.jpeg]
