# Supplementary material for: ZeMYB9 regulates cyanidin synthesis by activating the expression of flavonoid 3′-hydroxylase gene in Zinnia elegans
Source: Front Plant Sci. 2022 Oct 18;13:981086. doi: 10.3389/fpls.2022.981086 (PMC9623174; doi:10.3389/fpls.2022.981086)
Supplement: Supplementary Table 1 — Primer sequences for qRT-PCR. [file DataSheet_1.docx]

| **Table S1 Primer sequences for qRT-PCR.** | | |
| --- | --- | --- |
| **Primer name** | **Primer sequence (5' - 3')** | **Source** |
| ZeF3'H-qF | TTGCCCGTGACCCGAAAATGT | Qian et al., 2021 |
| ZeF3'H-qR | TTCGTCCAGCCCCAAAAGGTATGA |  |
| ZeMYB9-qF | ACCAGGAAGAACTGCTAATGATGT | This study |
| ZeMYB9-qR | TGTGACCGCTCGTGGTTGTTA |  |
| ZeGL3-qF | ACCGACGCCGAGTGGTATTTC | This study |
| ZeGL3-qR | TCTGCAAGGTGAGCATCGGAC |  |
| ZeACT-qF | TGCTCGTAGTCAAGAGCAACATATGCAAGC | Qian et al., 2021 |
| ZeACT-qR | CGTGACCTCACTGATTCCCTGATGAAAATC |  |
| PhCHS-A-QF | TTTGTTCGAGCTCGTTTCAGCAGCC | This study |
| PhCHS-A-QR | TGTAAGCCCAACTTCACGGAGATGGC |  |
| PhCHI-A-QF | ATACCTTGTTCCTTGCTGGTGCTGGG | This study |
| PhCHI-A-QR | ACTCCTGGGGGGTTTTGCCTTTCC |  |
| PhF3H-QF | CACCAGAGGCGATAGTGTATC | Zhang et al., 2021 |
| PhF3H-QR | GCAAGAATTTCCTCAATGGGC |  |
| PhF3'H-QF | TACCACCAGGTCCAAAACCATGGCC | Zhang et al., 2021 |
| PhF3'H-QR | CCATGGCTGCAGTTGATTGGTGTGG |  |
| PhF3′5′H-QF | GGAGCCATGCCACATGTTTCCTTAGC | Zhang et al., 2021 |
| PhF3′5′H-QR | GCCATGCCACATGTTCCAACTTTGAG |  |
| PhDFR-A-QF | GTGCCGACAGTTTGCGTCACTGGAGC | This study |
| PhDFR-A-QR | TTGGCAGTTCCAGCAGATGTTTCACC |  |
| PhANS-QF | TGAAAGCTTGGCTAAAAGTGGAATCC | This study |
| PhANS-QR | CGAAGATGTTTCCGATTCCATTCAAC |  |
| Ph3RT-QF | TCCTGCAAATGTTGATGTTTCAGCGG | This study |
| Ph3RT-QR | GCTGCTGTTGCACCCAACCTGAATG |  |
| Ph5GT-QF | TCTCACTTGCTCTGAAAATGGACAGC | Zhang et al., 2021 |
| Ph5GT-QR | AACTTCACGTGCTACCTCTGCTGCC |  |
| PhGST-QF | ACAGAGGGTCATGGTCTGCCTGATAG | This study |
| PhGST-QR | TGACAGGAACTTGTCCAAATGGCTG |  |
| PhEF1α-QF | CCTGGTCAAATTGGAAACGG | This study |
| PhEF1α-QR | CAGATCGCCTGTCAATCTTGG |  |
| NtCHS-QF | AAGCAAGAGAAACTAAAGGCTACAAG | Li et al., 2019 |
| NtCHS-QR | AAATCCAAAAAGCACACCCCAT |  |
| NtCHI-QF | CGGGTGCCTCCATTCTTTTTACT | Li et al., 2019b |
| NtCHI-QR | CCTGACACTCTTTCGGCGATACTAC |  |
| NtF3H-QF | CCAGACAAACCAGATGGATGGATAG | Li et al., 2019b |
| NtF3H-QR | CAAGGGTAAGGTCGGGCTGTG |  |
| NtF3'H-QF | TGGCTATTTCATTCCAAAAGGCTCA | Li et al., 2019b |
| NtF3'H-QR | CTTCAAAGTCATTTCCTCGCACATC |  |
| NtDFR-QF | GCAGTTGCTTCCCTTTTCTACC | Li et al., 2019b |
| NtDFR-QR | TTCCCCATTGGTTGACTTTCC |  |
| NtANS-QF | GTGCCTGGGTTACAACTTTTCTATG | Li et al., 2019b |
| NtANS-QR | CATTGCTTAGGATTTCAAGGGTGTC |  |
| NtAN1a-QF | ACCATTCTCGAACACCGAAG | Li et al., 2019b |
| NtAN1a-QR | TGCTAGGGCACAATGTGAAG |  |
| NtAN1b-QF | CTTGAACACTTCTCAAACCGA | Li et al., 2019b |
| NtAN1b-QR | TGCTAGGGCACAATGTGAAG |  |
| NtUFGT-QF | GAGTGCATTGGATGCCTTTT | Li et al., 2019b |
| NtUFGT-QR | CCAGCTCCATTAGGTCCTTG |  |
| NtEF1α-QF | TGGTTGTGACTTTTGGTCCCA | Li et al., 2019b |
| NtEF1α-QR | ACAAACCCACGCTTGAGATCC |  |

**Table S2 Primer sequences for promoter cloning.**

| **Primer name** | **Primer sequence (5' - 3')** |
| --- | --- |
| ZeF3'H-FSP1 | TGACCCAATTTTACTGTTGAGTTTCCGGCACTG |
| ZeF3'H-FSP2 | GTAAACTACAATTACAAACCTGACGAACATGACGG |
| ZeF3'H-FSP3 | GTTGCAGAAGGTACAGTAAGAAAGCGGTTAG |

**Table S3 Primer sequences for vector construction.**

| **Primer name** | **Primer sequence (5' - 3')** |
| --- | --- |
| pGreen0800 II -ZeF3′Hpro-F | ggtcgacggtatcgataGTATCTCTACACAAGGCCATAATTGGGC |
| pGreen0800 II -ZeF3′Hpro-R | ccgctctagaactagtgTTTATGTATGGGGGTTGAGACTTGTG |
| pAbAi-ZeF3'H-PRO-F | gtacccggggatctgtcgacTATCCCTCGGTTACTTATTAAAAGATATTGAAATTC |
| pAbAi-ZeF3'H-PRO-R | gcacatgcctcgaggTTTATGTATGGGGGTTGAGACTTGTGAGTG |
| pCNHP-eYFP-ZeMYB9-F | tgcccaaattcgcgccATGATCAGACCAAATGGTAATACGAGCT |
| pCNHP-eYFP-ZeMYB9-R | ctcgcccttgctcacTGAATCAGGAATGTCCCATATTGCATCATCTATAGGGA |
| pCNH-Flag-ZeMYB9-F | tgcccaaattcgcgccATGATCAGACCAAATGGTAATACGAGCT |
| pCNH-Flag-ZeMYB9-R | cgtcgtccttgtaatcTGAATCAGGAATGTCCCATATTGCATCATCT |
| pGBKT7-ZeGL3-F | tatggccatggaggccgaattcATGGGTACAAAAGAACACTTAAGGAGAAAATT |
| pGBKT7-ZeGL3-R | cgacggatccccgggttaAACTTTATGATGTGGAGCATCAAAGACTAAAG |
| pGADT7-Ze4243-2-F | ccatggaggccagtgaattcATGATCAGACCAAATGGTAATACGAGCT |
| pGADT7-Ze4243-2-R | tgcccacccgggtggctaTGAATCAGGAATGTCCCATATTGC |
| pCNHP-eYFP-ZeGL3-F | tgcccaaattcgcgccATGGGTACAAAAGAACACTTAAGGAGAAAAT |
| pCNHP-eYFP-ZeGL3-R | ctcgcccttgctcacATGCCTACTACCAATTACTCTCTGAAGT |
| pCNHP-cEYFP-ZeMYB9-F | tgcccaaattcgcgccATGATCAGACCAAATGGTAATACGAGCT |
| pCNHP-cEYFP-ZeMYB9-R | agctgcacgctgcctGAATCAGGAATGTCCCATATTGCATCATCT |
| pCNHP-nEYFP-ZeGL3-F | tgcccaaattcgcgccATGGGTACAAAAGAACACTTAAGGAGAAAAT |
| pCNHP-nEYFP-ZeGL3-R | ctcgcccttgctcacATGCCTACTACCAATTACTCTCTGAAGT |
| pCAMBIAI1300-CLuc-ZeMYB9-F | cggggcggtacccggATCAGACCAAATGGTAATACGAGCTC |
| pCAMBIAI1300-CLuc-ZeMYB9-R | cgaaagctatgcaggtcgacctaTGAATCAGGAATGTCCCATATTGCATCATCTATAG |
| pCAMBIAI1300-NLuc-ZeGL3-F | gagctcggtacccgGGATCCATGGGTACAAAAGAACACTTAAGGAGAAAATTG |
| pCAMBIAI1300-NLuc-ZeGL3-R | gcgtacgagatctggtcgacATGCCTACTACCAATTACTCTCTGAAGTG |
